# Identification and Characterization of GABAergic Projection Neurons from Ventral Hippocampus to Amygdala 

**DOI:** 10.3390/brainsci5030299

**Published:** 2015-07-31

**Authors:** Robert Lübkemann, Judith Eberhardt, Friedrich-Wilhelm Röhl, Kathrin Janitzky, Sven Nullmeier, Oliver Stork, Herbert Schwegler, Rüdiger Linke

**Affiliations:** 1 Institute of Anatomy, Otto-von-Guericke University, Leipziger Str. 44, D-39120 Magdeburg, Germany; E-Mails: robertluebkemann@hotmail.de (R.L.); judith.eberhardt@st.ovgu.de (J.E.); sven.nullmeier@med.ovgu.de (S.N.); herbert.schwegler@med.ovgu.de (H.S.); 2 Institute of Biometry and Medical Informatics, Otto-von-Guericke University, Leipziger Str. 44, D-39120 Magdeburg, Germany; E-Mail: friedrich-wilhelm.roehl@med.ovgu.de; 3 Clinic of Neurology, Otto-von-Guericke University, Leipziger Str. 44, D-39120 Magdeburg, Germany; E-Mail: kathrin.janitzky@med.ovgu.de; 4 Institute of Biology, Department of Genetics and Molecular Neurobiology, Otto-von-Guericke University, Leipziger Str. 44, D-39120 Magdeburg, Germany; E-Mail: oliver.stork@ovgu.de; 5 Center of Behavioral Brain Science, D-39120 Magdeburg, Germany

**Keywords:** fear, memory, GABAergic, hippocampus, amygdala

## Abstract

GABAergic local circuit neurons are critical for the network activity and functional interaction of the amygdala and hippocampus. Previously, we obtained evidence for a GABAergic contribution to the hippocampal projection into the basolateral amygdala. Using fluorogold retrograde labeling, we now demonstrate that this projection indeed has a prominent GABAergic component comprising 17% of the GABAergic neurons in the ventral hippocampus. A majority of the identified GABAergic projection neurons are located in the stratum oriens of area CA1, but cells are also found in the stratum pyramidale and stratum radiatum. We could detect the expression of different markers of interneuron subpopulations, including parvalbumin and calbindin, somatostatin, neuropeptide Y, and cholecystokinin in such retrogradely labeled GABA neurons. Thus GABAergic projection neurons to the amygdala comprise a neurochemically heterogeneous group of cells from different interneuron populations, well situated to control network activity patterns in the amygdalo-hippocampal system.

## 1. Introduction

The amygdala and hippocampus are tightly connected both anatomically and functionally. The ventral hippocampus has reciprocal connections with the basolateral amygdala [1,2] and both regions closely interact, *e.g.*, in the formation and expression of emotional memories [3,4,5]. GABAergic neurons are thought to play a pivotal role in the formation and retrieval of aspects of such emotional memories and the underlying network activity patterns in the amygdalo-hippocampal system [6,7,8].

**Figure 1:**
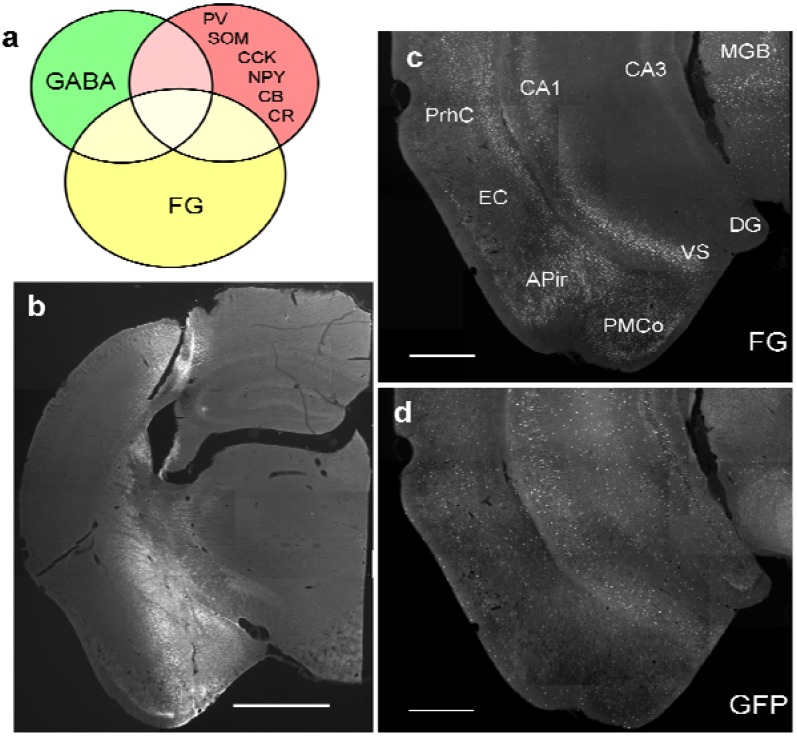

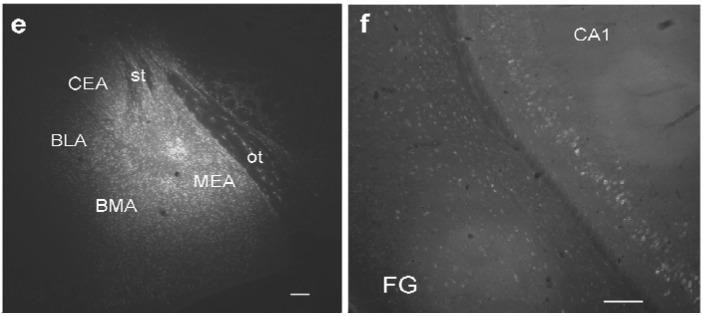
Study design and distribution of retrogradely labeled GABA neurons in the ventral hippocampus. (**a**) Schematic diagram of intersections of retrogradely labeled neurons, GABAergic neurons, and GABAergic markers in the ventral hippocampus; (**b**) typical site of pressure injection into the amygdala (case #382; see Table 1) at the site of maximal width. Cortical areas, *e.g.* the piriform cortex are largely spared; (**c**) Distribution of retrograde FG-labeling in the ventral hippocampus (#382). Labeling in the hippocampus is confined mainly to the pyramidal layer of CA1 and ventral subiculum (VS). Additional labeling is seen in the perirhinal cortex (PrhC), the amygdalo-piriform transition area, and the medial geniculate body (MGB). Rather sparse labeling is found in the entorhinal cortex (EC) and the posteromedial cortical nucleus of the amygdala (PMCo). Molecular layers of CA1 and VS and entire CA3 and DG regions are devoid of retrogradely labeled neurons; (**d**) Distribution of GFP-positive neurons in the same section; (**e**) maximal distribution of FG after an iontophoretic injection into the medial amygdala (MEA). Note the circular necrotic core (location of the needle tip) and the circular halo around the core, which is considered the active uptake zone. Beyond the halo is a circular diffusion zone. Note that the central nucleus (CEA), the basolateral nucleus (BLA), and the basomedial nucleus (BMA) are not reached by the halo but only by the diffusion zone. The optic tract (ot) medial to the injection limited the circular diffusion of tracer; (**f**) Retrograde labeling pattern after the iontophoretic injection seen in (**e**). Calibration bars = 1mm (**b**); 500μm (**c**,**d**); 100 μm (**e**, **f**).

GABAergic neurons are important local elements in the circuitry of the brain and are thought to shape the response properties of long-range projection neurons. During the last three decades, however, a growing body of evidence has accumulated that cortical GABAergic neurons also contribute to long-range projections, *e.g.*, in the septo-hippocampal system [9,10,11,12], between hippocampus and entorhinal cortex [13] and within the hippocampal formation [14,15], as well as within the neocortex [16,17]. In fact, we have recently demonstrated a GABAergic projection from the ventral hippocampal formation to the amygdala [18]. To further characterize this projection, in the current study we analyzed the distribution in the ventral hippocampus of GABAergic neurons retrogradely labeled from the amygdala and their expression of subpopulation-specific neurochemical markers, *i.e.* the calcium-binding proteins parvalbumin, calbindin, and calretinin and the neuropeptides NPY, somatostatin, and cholecystokinin (Figure 1a, Table 1) in GAD67-GFP knock-in mice.

An important role for amygdalo-hippocampal circuits in fear expression and extinction of fear memory was shown [19]. Anxiety disorders such as post-traumatic stress disorder (PTSD) produce changes in neuronal activity in these circuits [20]. Neuronal activation in the amygdala was decreased during extinction training of PTSD patients as compared to control patients and deficits in the anterior hippocampus (homologue to the rodents’ ventral hippocampus) were also shown in these patients [20,21]. GABAergic projection neurons of the ventral hippocampus to the amygdala can be expected to critically contribute to these processes. We here began with their anatomical and neurochemical characterization, which is an important prerequisite for a consecutive functional characterization.

**Table 1:** List of injected animals, GABAergic markers, and number of sections analyzed.

Injection Case #	PV	CB	CR	NPY	SOM	CCK
181	-	-	3	-	3	-
231	-	-	-	-	3	-
342	3	-	-	3	-	-
360	3	-	-	1	-	-
382_l	-	3	3	-	3	-
382_r	-	3	3	-	-	-
386_l	3	-	-	3	-	-
386_r	3	-	-	3	-	-
399_l	-	3	4	-	3	-
399_r	-	3	3	-	3	-
401	-	2	3	-	3	-
411	3	-	-	3	-	-
510_r	-	2	3	-	3	-
510_l	-	2	-	-	-	-
800	3	-	-	3	-	-
1512	-	-	-	-	-	3
1523	-	-	-	-	-	3
1522	-	-	-	-	-	-

PV, parvalbumin; CB, calbindin; CR, calretinin; NPY, neuropeptide Y; SOM, somatostatin; CCK, cholecystokinin.

## 2. Experimental Section

### 2.1. Animals

Twenty-seven heterozygous GAD67-GFP knock-in mice (14 males, 13 females) [22] aged 3–5 months were used for this study. These animals express green fluorescent protein (GFP) in GABAergic cells, allowing for a sensitive detection of retrogradely-labeled GABAergic cells. Mice were bred in our facility as heterozygotes and genotyped shortly after weaning [23]. All experiments were carried out in accordance with the European Council Directive (2010/63/EU) and the German Law on the Protection of Animals. All protocols were approved by the local council of animal care (Az. 42502-2-813 UniMD, Az. 42502-2-1130 UniMD).

### 2.2. Surgery

**Figure 2:**
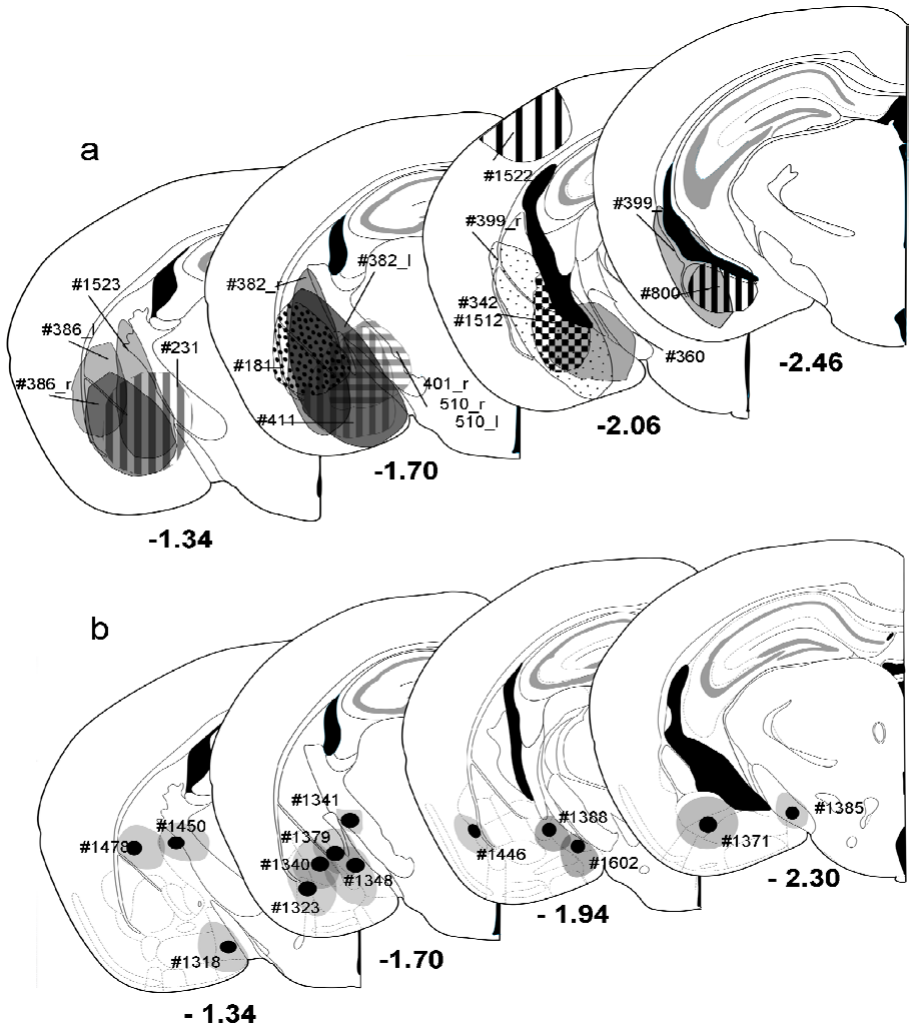
Summary of all injections made into mouse amygdala. (**a**) Pressure injections. Individual injections are placed into atlas drawings where the necrotic core was found and were labeled by different patterns. Rostral and caudal extensions were not incorporated. Each injection was labeled by case number. Numbers below sections indicate the distance from bregma; (**b**) Iontophoretic injections. Shown are the location of the necrotic core (black circle) and the largest extent (gray circle) of individual injections. Numbers beneath the injections are case numbers. Numbers below sections indicate the distance from bregma. Drawings adapted from Franklin and Paxinos [24].

Mice were anesthetized with a cocktail (0.1mL/10g body weight i.p.) of Ketamin (10 mg/mL) and Xylazine (1 mg/mL). The retrograde tracer Fluorogold (FG, Fluorochrome, Englewood, CO, USA; 4% in distilled water) was injected either using a Hamilton syringe or by means of iontophoresis into the amygdala. Using a 1-μL Hamilton^®^ syringe, FG was injected unilaterally (*n* = 9) or bilaterally (*n* = 4) into the amygdala (anterior-posterior coordinates (AP) −1.8, medio-lateral coordinates (ML) ± 2.8, dorso-ventral coordinates (DV) −4). Because the projection is strictly ipsilateral, each injection of those four bilaterally injected animals was treated as a single case. This procedure revealed 17 successful injection cases into the amygdala (cf. Figure 2a, Table 1). In order to label as many hippocampal cells as possible, large injections comprising 50 nL of tracer were made in order to cover the entire amygdala (Figure 1b). One injection was made into the cortex dorsal of the striatum and the amygdala to determine a potential contribution of cortical areas in hippocampal labeling (#1522, Figure 2a). To further test for specificity of labeling, small injections aimed at single nuclei of the amygdala (*n* = 13, Figure 1e, Figure 2b) were made iontophoretically using a precision current source (Stoelting, Woods Dale, IL) and applying the same coordinates as for the injections with the Hamilton syringe. A positive current of 2 μA was applied for 10–15 min at an interval cycle of 7 s on/off. Micropipettes had tip diameters between 30–50 μm. Stereotaxic coordinates were determined using the atlas of Franklin and Paxinos [24]. AP and ML values were determined from bregma and the midline suture of the skull, respectively. DV value was determined from the surface of the brain.

After a survival time of 7–14 days, mice received a lethal dose of barbiturate (Narcoren, Merial, Halbergmoos, Germany) and were transcardially perfused with 30 mL 0.9% saline, followed by 200 mL of a mixture of 4% paraformaldehyde and 15% saturated picric acid in 0.1M phosphate buffer pH 7.4 (PB). Brains were dissected from the skull and postfixed for an additional 5 h at room temperature. All brains were shock-frozen at −40 °C in isopropanol and stored at −80 °C until cutting.

### 2.3. Immunohistochemistry

Three to four series of coronal frozen sections (40 μm) were collected in cold PB. For chemical characterization of subpopulations of GFP-positive neurons, a series of sections was subjected to immunohistochemistry against either calcium-binding proteins parvalbumin (PV, SWant, Bellinzona, Switzerland; 1:5000; catalogue No 235), calbindin-D28k (CB, SWant, Bellinzona, Switzerland; 1:5000 mouse, Catalogue No 300) and calretinin (CR, SWant, Bellinzona, Switzerland, 1:5000, catalogue No 7696), and neuropeptides somatostatin (SOM, Santa Cruz, Santa Cruz, CA, USA; 1:400; catalogue No SC-7819)), neuropeptide Y (NPY, GeneTex, San Antonio, TX, USA; 1:16000; catalogue No GTX-10980), and Cholecystokinin (CCK, Immunostar, Hudson WI, USA; 1:1000, catalogue No 20078). Sections were incubated in the primary antibody for 2 days at 4 °C, washed in PB, and incubated in the respective secondary biotinylated antibody (1:200; for PV, CB and CR: biotinylated anti-mouse IgG, for SOM and CCK biotinylated anti-rabbit IgG, for NPY biotinylated anti-goat IgG, Vector Laboratories, Burlingame, CA, USA). The peptides were visualized with avidin-Cy3 (1:1000 in 0.1M PB pH 7.4, Jackson Laboratories, West Grove, PA, USA). Stained sections were mounted and coverslipped with Mowiol (Hoechst, Mannheim, Germany). For each antibody, we included a negative control section by omitting the primary antibody during incubation. In all cases, this resulted in a lack of staining.

### 2.4. Analysis

Sections of the ventral hippocampus were analyzed with a Zeiss Axioplan microscope equipped with fluorescence optics using appropriate filter sets for FG (Zeiss 01: BP 365/12, FT 395, LP 397; Zeiss 49: G 365, FT395, BP445/50), GFP (Zeiss 10: BP 450-490, FT 510, BP515-565), and Cy-3 (Zeiss 15: BP546/12, FT580, LP590). A photomontage at low magnification (100×) from the region of interest (ROI; ventral CA1 and the transition to the ventral subiculum as determined by retrograde labeling, see Figure 1c,d) was constructed in GIMP (GNU Image Manipulation Program, 2.6) for FG-labeling, GFP-labeling, and Cy-3 labeling for each series, respectively. To standardize the area, the upper border of ROI was arbitrarily defined by a horizontal line drawn through the perirhinal sulcus. In the congruent photomontages, boundaries of hippocampal layers were determined and mapped to a separate plane of GIMP. Due to the lack of a clear detectable boundary, the fiber layer superficial to the pyramidal neurons was collectively referred to as the molecular layer. The distribution of labeled cells was plotted for each color into separate planes of GIMP using different symbols. Superimposing the various planes of plotted neurons revealed prospective double- and triple-labeled neurons. Doubtful potential double- and triple labeled neurons were re-examined in the original sections using high magnification (400×) to confirm results obtained from low magnification microscopy. As a rule this procedure was performed for three sections per injection. In some cases only one or two sections per series were available due to the loss of sections. The percentages of double- and triple labeled neurons were calculated for each section and afterwards averaged to reduce measurement errors. For three cases of calbindin, only two sections in the series were available and for one case of NPY only one section could be analyzed (see Table 1). Sections were spaced by at least 120 μm. During the mapping procedure all mapped neurons were counted. For each injection case up to three GABAergic marker proteins could be tested (cf. Table 1). That is, that for each GABAergic marker the numbers of retrogradely labeled neurons, of GABAergic neurons and of double- and triple labeled neurons were determined by counting all neurons in the series of sections and calculating the mean.

To determine the numbers of retrogradely labeled neurons, GFP-labeled neurons, and double-labeled neurons per injection case, the means of the counted numbers of the GABAergic marker cases were averaged. This gives the average number of GABAergic projection neurons for a single injection. Using these average numbers and the average numbers of single labeled neurons (FG and GFP, respectively), the percentage of GABAergic projection neurons was calculated for the population of retrogradely labeled neurons and the population of GABAergic neurons (Table 2).

Then, percentages of the GABAergic marker proteins within the population of double-labeled GABAergic were determined using the averages of double-labeled neurons (from injection cases set as 100%) and the averages of triple-labeled neurons determined from GABAergic marker cases (Table 4a,b).

Due to the rather large injections, a contamination with tracer of the overlying cortex and striatum was always found. Spillover of tracer into the piriform cortex, a target of the ventral hippocampus [25] that borders the amygdala laterally, was extremely rare. Injections into the overlying striatum or cortex did not reveal any retrograde labeling in the ventral hippocampus. For correlating the different injection sites and sizes to the number of retrogradely labeled neurons, the volume of the injection site within the amygdala was estimated. Starting from the section with the largest extent of the injection site in the amygdala, the area of diffusion of the tracer in both two sections rostral and caudal of the starting section was measured using formula V = A1×h + A2×h + A3×h + A4×h + A5×h (V = Volume, A = area of diffusion of tracer within the amygdala, h = distance between sections, 1–5 = number of section). Area of tracer diffusion was measured using the calibrated Zeiss Axioplan software (Version 4.8; Zeiss, Oberkochen, Germany). Correlation was tested with the non-parametric Spearman rank test. The results should be interpreted in an exploratory manner. Statistical analyses were carried out with SAS^®^ Version 9.3 (SAS Institute Inc., Cary, NC, USA).

## 3. Results

Pressure injections of FG into the amygdala (Figure 1b, Figure 2a) resulted in retrograde labeling of neurons in the ventral hippocampal formation, the amygdalo-piriform transition area, the perirhinal cortex, and the paralaminar thalamus close to the medial geniculate body (Figure 1c). Within the hippocampal formation, retrogradely labeled neurons were found mainly in the stratum oriens and the pyramidal layer of ventral CA1 and the adjacent subiculum (Figure 1c). Few neurons in the CA1 molecular layer close to the pyramidal layer were also labeled. No retrograde labeling was observed in the dorsal hippocampus, in the CA3 or dentate gyrus of the ventral hippocampus, or in the contralateral hemisphere, unless the injection needle had accidentally injured the fimbria/fornix bundle.

**Figure 3:**
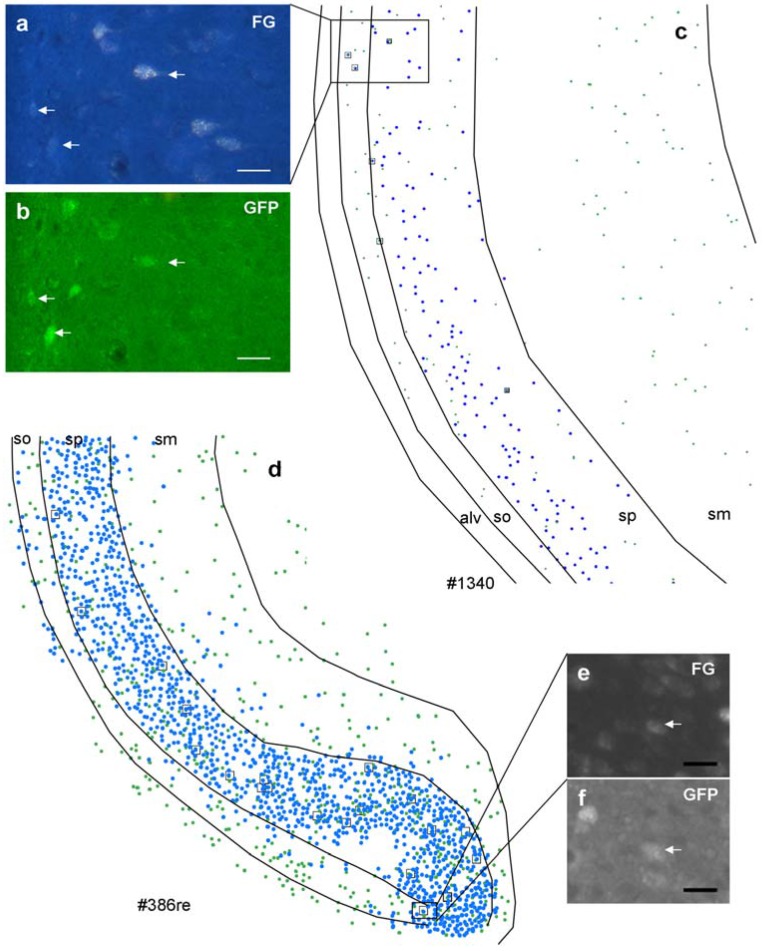
Distribution of retrogradely labeled (blue spots) and GABAergic neurons (green spots) in the ventral hippocampus after an iontophoretic (**c**) and after a pressure injection (**d**). Case numbers of injections are indicated below the chart. Note that the distribution pattern is identical after both injections; however, numbers of retrogradely labeled neurons differ dramatically. In the boxed areas, retrogradely-labeled GABAergic neurons were shown ((**a**), FG; (**b**), GFP after iontophoretic injection; (**e**), FG; (**f**), GFP after pressure injection). Note that retrogradely labeled neurons are also variably labeled. Calibration bars = 20 μm ((**a**), (**b**), (**e**), and (**f**)).

Iontophoretic injections of FG into the amygdala (Figure 1e, Figure 2b) covered a much smaller area within the amygdala and labeled fewer neurons in the hippocampus than the pressure injections (Figure 1f). However, the overall labeling pattern within the hippocampus, *i.e.* the exclusive labeling in the ventral CA1 and subiculum, was exactly the same. Figure 3 illustrates the distribution of retrogradely labeled neurons and GFP-positive neurons after an iontophoretic injection (Figure 3b, #1340) and for a pressure injection (Figure 3a, #386_r).

The small insets demonstrate a double labeled cell in the boxed area, respectively. However, numbers of labeled neurons in the hippocampus depend strongly on the location of the injection within the different nuclei of the amygdala (Table 2), with the largest numbers in the basolateral and basomedial nucleus.

**Table 2:** Location of iontophoretic injections and retrogradely-labeled neurons.

Case #	Injection Site	# Retrogradely-Labeled Neurons	# of Double- Labeled Neurons
#1323	BLv	42	0
#1371	BMp	532	7
#1318	MePD	129	0
#1388	MePD	195	4
#1602	MePD	62	0
#1348	MePD/BM	345	1
#1340	BLA/BMP	239	6
#1379	CeL	42	0
#1478	CPu	4	0
#1450	GP/ic	0	0
#1341	ic	0	0
#1446	VEn	7	0
#1385	cp	0	0

Number of retrogradely-labeled neurons and number of double labeled neurons per section in the ventral hippocampus after iontophoretic injections of FG into different nuclei of the amygdala. BLv, basolateral nucleus of the amygdala ventral part; BLA, basolateral nucleus of the amygdala, anterior part; BM, basomedial nucleus of the amygdala (P, posterior part); CeL, central nucleus of the amygdala, lateral part; cp, cerebral peduncle; CPu, striatum; GP, Globus pallidus; ic, internal capsule; MePD, medial nucleus of the amygdala, posterodorsal part; VEn, ventral endopiriform nucleus.

**Figure 4:**
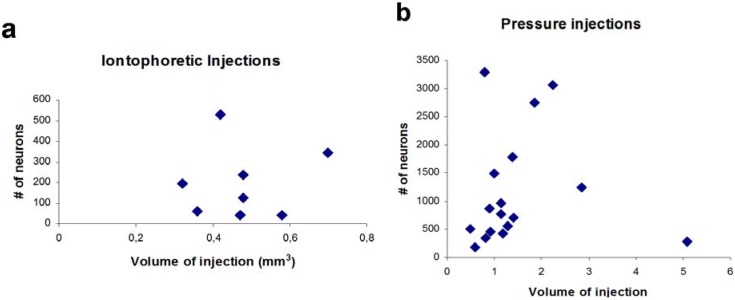
Correlation between numbers of retrogradely labeled neurons in the ventral hippocampus and size of the iontophoretic injection within the amygdala. (**a**) Correlation for iontophoretic injections (*n* = 8). Only those injections are considered that hit the amygdala. Injections that did not hit the amygdala revealed no retrogradely labeled neurons in the hippocampus; (**b**) Correlation for pressure injection cases (*n* = 17, cf. Table 1, Figure 2a).

Interestingly, the volume of injection sites and the number of labeled neurons did not correlate (*r* = −0.024, *p* > 0.05, *n* = 8, Figure 4). Due to the very low numbers of GABAergic projection neurons in these cases (0–7 per section), we returned to pressure injections, which filled the entire amygdala, for quantitative analysis.

Due to the differences in pressure injections (size, location, transport), absolute numbers of retrogradely labeled neurons varied between individual animals (see Table 3). However, the volume of the injection site in the amygdala and the numbers of retrogradely labeled neurons in the region of interest correlated only weakly (*r* = 0.24, *p* > 0.05, *n* = 17, Spearman rank correlation), indicating that adjacent areas overlying the injection (*e.g.* striatum, cerebral cortex) did not receive a substantial projection from the ventral hippocampus. Iontophoretic injections that were located within the striatum (Figure 2b, #1478, #1450) or an injection into the cerebral cortex at the penetration site of the Hamilton syringe (Figure 2a, #1522) revealed no labeling in the ventral hippocampus. This was supported by anterograde labeling studies [2,18,26]. The only area that received a substantial projection from the ventral hippocampus was the piriform cortex [25] but contamination of this area was avoided.

GFP-labeled neurons were more or less uniformly distributed in the ventral hippocampal area (Figure 1c), although their numbers show wide differences between individuals (Table 3). In all cases a significant contribution of GABAergic cells to the projection could be detected, with retrogradely-labeled GABAergic neurons frequently observed in stratum oriens and stratum pyramidale and rarely in stratum moleculare. Cell counts revealed that an average of 5% of the total number of retrogradely labeled cells was also positive for GFP (Table 3) and in turn that 16% of all GFP-positive neurons in the ventral hippocampus were retrogradely labeled with FG (Table 3).

**Table 3:** Average of counts of retrogradely-labeled, GABAergic, and double-labeled neurons and the percentages of GABAergic projection neurons from either population. The number of series/injection is derived from Table 1, because in some animals three series were used and in others only one. At least three sections were analyzed for each case. In cases where more than one series (most often three sections/series; see Table 1) was analyzed for one injection case, the mean of each series was averaged to obtain the mean of the case.

Injection Case #	Number of series /injection	# of retro-gradely-labeled neurons /injection	# of GABA-ergic neurons /injection	# of double-labeled neurons / injection	% double-labeled neurons from retrogradely labeled neurons	% double-labeled neuron from GABAergic neurons
181	2	505.5	183.5	52.8	10.45	28.77
231	3	342	112	6.3	1.84	5.63
342	2	863.9	126.2	18.5	2.14	14.66
360	2	1781.4	235.5	35.4	1.99	15.03
382_l	3	421.1	262.5	3	0.71	1.14
382_r	2	764.3	261.7	68.2	8.92	26.06
386_l	2	271.8	242	15.4	5.67	6.36
386_r	2	1238.2	289	48.4	3.91	16.75
399_l	3	696.6	261	27.7	3.98	10.61
399_r	3	971.1	248.7	64.1	6.60	25.77
401	3	1485.6	424.6	93.8	6.31	22.09
411	2	2744.4	315.4	81	2.95	25.68
510_r	3	463.6	195.9	26.9	5.80	13.73
510_l	1	175	156	18	10.29	11.54
800	2	3058.4	725.5	83.7	2.74	11.54
1512	1	3292	597.3	153	4.65	25.62
1523	1	548.3	645.7	62.7	11.44	9.71
Mean		1154.31	310.74	50.52	5.32	15.92
SEM		241.88	44.06	9.37	0.8	2.03

To further classify the GABAergic neuron population that contributes to this projection (see Figure 1a, Table 1), we next stained cells for calcium binding proteins and neuropeptides known to be expressed specifically in subpopulations of hippocampal GABAergic neurons [27]. The expression pattern of these markers was found to match with previous observations (*e.g.* [28]).

**Figure 5:**
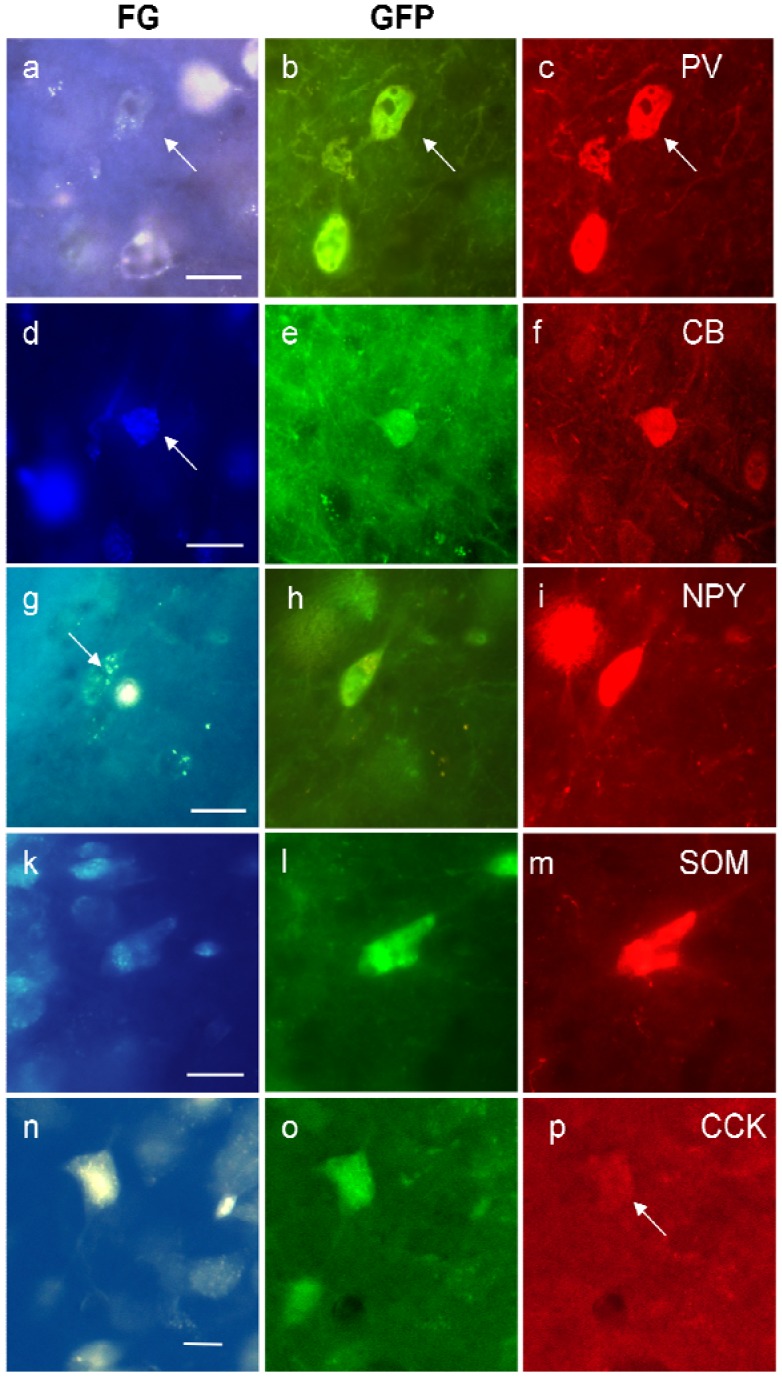
Examples of triple-labeled neurons in the ventral hippocampus for FG, GFP, and parvalbumin (PV, (**a**)–(**c**)); calbindin (CB, (**d**)–(**f**)); neuropeptide Y (NPY, (**g**)–(**i**)); somatostatin (SOM, (**k**)–(**m**)) and cholecystokinin (CCK, (**n**)–(**p**)). The white spot in (**g**) is an artifact. Calibration bars = 20 μm.

We regularly detected the calcium-binding proteins PV or the neuropeptides NPY, CCK, and SOM in FG/GFP double-labeled cells (Figure 5). CB-projecting GABAergic neurons were rare and CR-projecting GABAergic neurons were virtually absent (see Table 4). Our results show that the majority of GABAergic projection neurons appear to contain PV, followed by NPY and CCK (see Table 4).

Figure 5 demonstrates triple-labeled neurons for each peptide, respectively. Although rare in general, triple-labeled neurons were found most often in the stratum pyramidale, followed by stratum oriens.

**Table 4:** (**a**) Mean numbers of GABAergic projection neurons that were labeled for different GABAergic markers; (**b**) percentages of GABAergic projection neurons that were labeled for different GABAergic markers.

(a)							
Injection Case #	Mean FG + GABA /injection	Mean FG + GABA + PV	Mean FG+ GABA + NPY	Mean FG + GABA+ CCK	Mean FG + GABA + CB	mean FG + GABA + CR	Mean FG + GABA + SOM
181_r	52.8	-	-	-	-	0.33	5.67
231_r	6.3	-	-	-	-		0
382_l	2.99	-	-	-	0	0	0
382_r	68.17	-	-	-	3	0.3	-
399_l	27.67	-	-	-	4	0.5	0
399_r	64.11	-	-	-	1.67	1	3.3
401_r	93.76	-	-	-	3	2.67	10
510_l	18	-	-	-	1	-	-
510_r	26.9	-	-	-	1.5	0	2
342	18.5	16.3	1.7	-	-	-	-
386_r	48.4	11.7	8.3	-	-	-	-
386_l	15.4	1.3	3.7	-	-	-	-
411	81	16	11.7	-	-	-	-
360_r	35.4	9.3	2	-	-	-	-
800	83.7	14.3	27	-	-	-	-
1512	153	-	-	9.3	-	-	-
1523	62.7	-	-	11.7	-	-	-
**(b)**							
**InjectionCase #**	**Mean FG + GABA /injection**	**% FG+ GABA + PARV of FG+GABA**	**% FG + GABA + NPY of FG + GABA**	**% FG + GABA +CCK of FG + GABA**	**% FG + GABA +CB of FG + GABA**	**% FG + GABA + CR of FG + GABA**	**% FG + GABA + SOM of FG + GABA**
81_r	52.8	-	-	-	-	0.6	10.7
231_r	6.3	-	-	-	-		0
382_l	2.99	-	-	-	0	0	0
382_r	68.17	-	-	-	4.4	0.4	
399_l	27.67	-	-	-	14.4	1.8	0
399_r	64.11	-	-	-	2.7	1.6	5.2
401_r	93.76	-	-	-	3.2	2.9	10.7
510_l	18	-	-	-	5.6		
510_r	26.9	-	-	-	5.6	0	7.4
342	18.5	88	9.2	-	-	-	-
386_r	48.4	24.2	17.1	-	-	-	-
86_l	15.4	8.4	24	-	-	-	-
411	81	19.8	14.4	-	-	-	-
360_r	35.4	26.3	5.6	-	-	-	-
800	83.7	17.1	32.3	-	-	-	-
1512	153	-	-	6.1	-	-	-
1523	62.7	-	-	18.7	-	-	-
Mean		30.63	17.10	12.40	5.13	1.04	4.86
SEM		11.76	4.00	6.30	1.71	0.41	1.86

## 4. Discussion

GABAergic interneurons of the hippocampus comprise morphologically, physiologically, and neurochemically different subpopulations [29]. They control the excitability and activity flow in the hippocampus as well as the generation of local network oscillations, thought to be the basis of cognitive and emotional information processing [28,30]. Here we show that approximately 16% of all GABAergic neurons in the hippocampal CA1/subiculum transition zone project to the amygdala, thus comprising about 5% of all retrogradely labeled neurons from the amygdala in this region. GABAergic projection neurons to the amygdala are differentially distributed within the hippocampal layers and include at least five subpopulations of calcium-binding proteins (PV, CB) and neuropeptide-containing (NPY, CCK, SOM) neurons.

Immunohistochemistry and tracing techniques are generally rather qualitative methods. In order to obtain an estimation of the proportion of GABAergic cell populations contributing to the hippocampo-amygdalar projection, we performed single large injections of retrograde tracer into the amygdala and determined the relative cell numbers of traced interneurons in the ventral hippocampus. This type of injection procedure inevitably contaminated overlying cortical and striatal structures that may contribute to the overall projection. Small iontophoretic injections into the striatum or the overlying cortex, however, revealed no labeling in the ventral hippocampus, suggesting that these structures are not the target of the ventral hippocampus. This was supported by anterograde tracing studies [2,18,25,26]. Although Cenquizca and Swanson [25] report a small projection into cortical areas, these areas (gustatory, supplemental somatosensory, auditory) differ from those that were penetrated by the needle (primary somatosensory). Thus, we concluded that contamination of these areas is not critical to our count.

Small iontophoretic injections into single amygdala nuclei (Figure 1e, Figure 2b, Table 2) were found not to label a sufficiently large number of neurons for quantitative analysis. For example, injection into the central nucleus, the medial nucleus, or the ventral basolateral nucleus produced a pattern of retrograde labeling in the ventral hippocampus that was completely comparable to our large injections. However, we considered the numbers of 62–195 labeled neurons and 0–4 double-labeled neurons per section that could be achieved with those small injections as insufficient to reliably evaluate subpopulations of GABAergic projection neurons. Injections into the basomedial and basolateral nuclei revealed more neurons (Table 2), but even here the number of double-labeled neurons was considered too low to reliably search for neurochemical differences in these neurons. The sparse labeling or the often light labeling of hippocampal neurons after iontophoretic injections is not due to a transport problem, as indicated by the often intensely labeled neurons in the paralaminar thalamic nuclei medial to the medial geniculate body that target the amygdala [31,32]. Also, even in the hippocampus intensely labeled cells are found close to lightly labeled cells. This might be due to unequal access for axons to the tracer deposits.

A severe contamination of areas during pressure injections might be due to spillage of tracer into the piriform cortex which is situated lateral to the amygdala. This area receives a projection from the ventral hippocampus [25]. However, all our injections avoided the piriform cortex and leakage of tracer was thus considered minimal. Injections that hit the fornix on the medial side of the amygdala were generally excluded from the analysis. In the analyzed brains, all retrograde labeling was confined to the ipsilateral ventral hippocampus and thus specific for the hippocampo-amygdalar projection.

The current data confirm our previous observation of a GABAergic contribution to this projection [18]. The large contribution of retrogradely-labeled cells to some interneuron subpopulations, in particular to about 30% of PV+ neurons and approximately 17% and 12% of NPY- and CCK-containing neurons, suggests a significant contribution of these cell groups to long-range interactions within the amygdalo-hippocampal system.

Such an involvement in long-range interactions may be a more general feature, at least for some interneuron subpopulations in the hippocampus [33]. GABAergic projection neurons to the entorhinal cortex [13], the medial septum, and the retrosplenial/subicular area [34] also predominantly originate in the stratum oriens of area CA1. The question arises whether the hippocampo-amygdalar projection neurons identified in this study comprise a distinct class of GABAergic cells, or overlap with the previously identified populations. GABAergic projection neurons to the entorhinal cortex as well as a large proportion of cells projecting to the medial septum and subiculum were positive for somatostatin, whereas those projecting to the retrosplenial cortex were not [12,13,35]. A smaller proportion of cells projecting to subiculum and medial septum were positive for NPY, parvalbumin, and calbindin. Thus, these projection neurons display a similar neurochemical profile to those identified here both in terms of localization and neurochemical characteristics. We began to address this question further with combined injections into both septum and amygdala regions. These revealed retrogradely labeled neurons in CA1 and CA3 of the dorsal and ventral hippocampus and in the contralateral hippocampus (projecting to the septum) and ipsilateral in the ventral hippocampus (projecting to the amygdala), but no clear-cut double labeling of neurons was detected (Nullmeier and Linke, unpublished observations).

GABAergic neurons in the hippocampus are critically involved in the generation and propagation of rhythmic network oscillations [36,37], and hippocampal GABAergic long-range projection neurons have been suggested to coordinate and synchronize rhythmic activity patterns with other brain regions. For example, GABAergic cells projecting to both the septum and the retrohippocampal area were found to fire in phase with theta oscillations, while some of the SOM-positive double projection cells in the stratum oriens fired during the ascending phase of gamma oscillations. Indeed, optogenetic stimulation of long-range GABAergic axons in the reciprocal entorhino-hippocampal projection enhanced rhythmic activity in the theta frequency range in both target regions [13].

It is likely that GABAergic projection neurons to the amygdala are similarly bound to specific hippocampal activity patterns. This may be relevant, in particular, for the formation and retrieval of emotional memories. The amygdala and hippocampus tightly interact in the formation of episodic memories and their modulation according to salience. For example, the ventral hippocampus and amygdala both mediate the effects of glucocorticoids on fear memory [3] and both areas are involved in the expression of contextually conditioned fear [4,5]. The amygdala and hippocampus synchronize at theta frequencies during the retrieval of long-term fear memory [38,39]; this synchronization is dependent on GABAergic transmission: reduced GABA synthesis in mice deficient in the 65 kD isoform of glutamic acid decarboxylase leads to disturbances in theta synchronization between these areas, associated with generalization of auditory fear memory and disturbed fear extinction [6,7].

Prior studies showed that GABAergic mechanisms in the ventral hippocampus are involved in the mediation of context-specific fear memory retrieval after extinction [40]. Thus, understanding these processes has immediate clinical relevance, because a main symptom of PTSD is an exaggerated and persistent fear response to reminders of the traumatic event, and the current behavioral treatment of choice, exposure therapy, relies on extinction-based mechanisms that could be supported by selective drug therapy [20].

Jinno *et al.* [34] have reported that GABAergic double projection neurons have larger axons and thicker myelin sheets than non-GABAergic projection neurons, indicating that they may be able to relay information faster to downstream areas and thus, although few in number, may play a critical role in the long-range synchronization of network patterns. Our previous study demonstrated that projection neurons target both glutamatergic and GABAergic cells in the basal amygdala. Representing as many as 16% of the total number of GABAergic interneurons in the ventral hippocampus, they represent a considerable population of cells and are well suited to convey network activity patterns to the amygdala during different emotional states.
